# Heterogeneity of Microbiota Dysbiosis in Chronic Rhinosinusitis: Potential Clinical Implications and Microbial Community Mechanisms Contributing to Sinonasal Inflammation

**DOI:** 10.3389/fcimb.2018.00168

**Published:** 2018-05-23

**Authors:** Keehoon Lee, Steven D. Pletcher, Susan V. Lynch, Andrew N. Goldberg, Emily K. Cope

**Affiliations:** ^1^Department of Biological Sciences, Pathogen and Microbiome Institute, Northern Arizona University, Flagstaff, AZ, United States; ^2^Department of Otolaryngology Head and Neck Surgery, University of California, San Francisco, San Francisco, CA, United States; ^3^Division of Medicine, Department of Gastroenterology, University of California, San Francisco, San Francisco, CA, United States

**Keywords:** microbiome, microbiota, chronic rhinosinusitis, biofilm, interspecies interaction, microbiome-host interaction

## Abstract

Recent studies leveraging next-generation sequencing and functional approaches to understand the human microbiota have demonstrated the presence of diverse, niche-specific microbial communities at nearly every mucosal surface. These microbes contribute to the development and function of physiologic and immunological features that are key to host health status. Not surprisingly, several chronic inflammatory diseases have been attributed to dysbiosis of microbiota composition or function, including chronic rhinosinusitis (CRS). CRS is a heterogeneous disease characterized by inflammation of the sinonasal cavity and mucosal microbiota dysbiosis. Inflammatory phenotypes and bacterial community compositions vary considerably across individuals with CRS, complicating current studies that seek to address causality of a dysbiotic microbiome as a driver or initiator of persistent sinonasal inflammation. Murine models have provided some experimental evidence that alterations in local microbial communities and microbially-produced metabolites influence health status. In this perspective, we will discuss the clinical implications of distinct microbial compositions and community-level functions in CRS and how mucosal microbiota relate to the diverse inflammatory endotypes that are frequently observed. We will also describe specific microbial interactions that can deterministically shape the pattern of co-colonizers and the resulting metabolic products that drive or exacerbate host inflammation. These findings are discussed in the context of CRS-associated inflammation and in other chronic inflammatory diseases that share features observed in CRS. An improved understanding of CRS patient stratification offers the opportunity to personalize therapeutic regimens and to design novel treatments aimed at manipulation of the disease-associated microbiota to restore sinus health.

## CRS patient heterogeneity, a clinical problem

In medicine, human interactions with bacteria are primarily adversarial. Microbes are frequently detected by culture-based techniques developed in the 1800s. Detection of traditional bacterial pathogens often triggers an antibiotic onslaught with little understanding of, or regard for, collateral damage to commensal microbial species. While this approach is a critical and effective technique for the evaluation and treatment of acute bacterial infections, this algorithm offers little insight into or benefit for chronic inflammatory disorders associated with microbial dysbiosis.

Chronic Rhinosinusitis (CRS) is an inflammatory disorder of the paranasal sinuses. Although accurate data on the prevalence of CRS is difficult to find, surveys suggest that up to 15% of the US population suffers from symptoms of CRS (Collins, 1997; Blackwell et al., 2002). The quality of life impact from CRS is estimated to be similar to or greater than that of chronic obstructive pulmonary disease and congestive heart failure (Gliklich and Metson, 1995). In the United States, the overall economic burden for chronic sinusitis is estimated at $8.6 billion/year (Bhattacharyya, 2011).

This formidable disease burden has resulted in significant efforts to delineate the pathophysiology of CRS. Despite multiple proposed theories, no unifying etiology has emerged. Fungi (Ponikau et al., 1999), innate immune dysfunction (Lane, 2009), bacterial biofilms (Palmer, 2006), staphylococcal superantigens (Bachert et al., 2007), chronic osteitis (Kennedy et al., 1998), and genetic alterations of taste receptors (Carey et al., 2016) have all been implicated as potential etiologies for CRS. Further complicating these efforts, CRS is a clinically defined entity representing multiple pathologies or disease endotypes. Recent efforts have focused on defining these endotypes and have focused on biomarker-based categorizations with the potential to both improve our understanding of CRS pathophysiology and identify target patient sub-groups for specific biologic therapies. The classic pathophysiologic theories of CRS were developed prior to the advent of culture-independent bacterial detection and classification and, along with current endotype categorizations, appear to ignore or oversimplify the role of microbes within the sinuses.

Recent culture-independent evaluations of microbes in chronic rhinosinusitis demonstrate a collapse of microbial diversity in the sinuses of CRS patients relative to healthy controls (Abreu et al., 2012; Biswas et al., 2015) and suggest a mucosal protective role against pathogen infection by the native microbiota (Abreu et al., 2012). Overall, the relationship between these microbial communities and epithelial immune response is poorly understood. Comprehensive evaluation of sinus microbial communities in combination with local host immune response suggests a critical relationship at the interface of host and microbiota which may define health and disease in chronic rhinosinusitis (Cope et al., 2017).

## The microbiome in chronic airway disease

Advances in the field of human microbiome over the past two decades have highlighted the importance of host-associated microbiota in human health [Definitions in Box 1 (Lynch and Pedersen, 2016)]. Humans are host to a staggering number of microbes that contribute directly or indirectly to functions critical to maintaining human health such as immunity, metabolism, nutrient acquisition (Ichinohe et al., 2011; Lynch and Pedersen, 2016; Shukla et al., 2017). Recent estimates suggest that we harbor approximately 39 trillion bacterial cells, with the greatest burden in the colon (Sender et al., 2016). The diverse microbial communities that colonize the human host exhibit niche specificity and are compositionally distinct in different body habitats, including the GI tract, skin, mouth, and airways (Costello et al., 2009); perturbations (functional or compositional) to the microbiome at a specific body site are often hallmarks of disease (Abreu et al., 2012; Yu et al., 2015). Research that has largely focused on gastrointestinal (GI) microbiota-host interactions has demonstrated that features of the gut microbiome are causal in several diseases including obesity and Kwashiorkor (Smith et al., 2013). Moreover, microbial-derived products, including metabolites such as butyrate, regulate differentiation, and activity of immune cell populations such as CD4+FOXP3+ regulatory T cells (Furusawa et al., 2013) which are critical for down-regulation of inflammatory T-helper cell activities and contribute diminishing colitis and allergic diarrhea in a murine model (Atarashi et al., 2013). These results suggest that microbial metabolic products may be valuable as immunomodulatory therapeutics.

Box 1 **Definitions**

- Amplicon Sequencing: High-throughput sequencing of an amplified fragment of specific biomarker genes, e.g., 16S rRNA gene, 18S rRNA gene, internal transcribed spacer (ITS) gene, to determine microbiota composition.
- Metabolomics: Large-scale mass-spectrometry based detection of small molecules
- Metagenomics: High-throughput sequencing of the collective multi-species genomic content to determine microbial functional gene capacity.
- Metatranscriptomics: High-throughput sequencing of mRNA produced by a multi-species microbiome
- Microbiome: The collection of microbes (bacteria, fungi, and/or viruses) their genomes and interactions within a specific ecosystem.
- Microbiota: The collection of microorganisms that colonize a defined niche typically profiled by biomarker gene amplicon sequencing.

Our understanding of the role of respiratory tract microbiota in host health and disease remains nascent. Until recently, the respiratory tract of healthy individuals had been considered as a relatively sterile environment. Culture-independent evaluations have refuted this dogma; microbial communities are now known to be present in the upper (Abreu et al., 2012; Cope et al., 2017) and lower airways (Huffnagle and Dickson, 2015). Furthermore, respiratory microbial communities exhibit significant associations with respiratory health status. Changes in diversity or composition of the lung microbiota correspond to disease progression or severity (Huang et al., 2011, 2015; Goleva et al., 2013; Marsland and Gollwitzer, 2014; Cribbs and Beck, 2017). A recent study demonstrated significant differences in microbial composition between atopic asthma (*n* = 42), non-asthmatic atopic patients (*n* = 21), and healthy subjects (*n* = 21). Asthmatic subjects were enriched with *Haemophilus, Neisseria, Fusobacterium*, and *Porphyromonas* (Durack et al., 2017). Within asthmatics, responsiveness to the inhaled corticosteroid (ICS) fluticasone was related to features of the lung microbiome; the microbiota in ICS-responders were compositionally similar to healthy controls at baseline (Durack et al., 2017). Respiratory microbiota, including microbial-derived metabolites, represent key drivers of inflammatory responses in the airways. Metabolomic and 16S rRNA gene sequence analysis of bronchoalveolar (BAL) fluid of 39 HIV-infected and 20 HIV-uninfected individuals demonstrated 219 metabolic features in the lungs that correlated with peripheral blood CD4 T cell counts; 91 of these features were elevated in HIV patients. *Staphylococcaceae, Norocardiaceae*, and *Streptococcaceae* were positively correlated with HIV-metabolic signatures (Cribbs et al., 2016). This supports a recent study that examined lung microbiota and host immune response in a large cohort of HIV patients with pneumonia (*n* = 182). Three discrete lung microbiome compositional states (MCS) were related to patient outcomes, including mortality. The MCS that conferred an increased risk for mortality was characterized by elevated pulmonary T_H_2 cytokine gene expression (Shenoy et al., 2016). These findings emphasize the need for a new perspective on the role of the airway microbiome when considering both the origins of chronic airway disease development and the role of the airway microbiome in established disease.

In the first year of life, the frequency of acute respiratory infections drives upper airway microbial composition and impacts the development of childhood asthma (Biesbroek et al., 2014a; Teo et al., 2015). Interpersonal variation in early-life microbiota composition is relatively high and influenced by a range of factors including birth mode (Rodríguez et al., 2015; Bosch et al., 2016), breastfeeding (Azad et al., 2013; Biesbroek et al., 2014b), antibiotic use (Leclercq et al., 2017), and lung function (Teo et al., 2015). A prospective study of 234 children sampled during the first week of life, at 2, 6, and 12 months of age demonstrated nasopharyngeal communities undergo rapid niche specification in the first year of life (Bosch et al., 2016). By 1–2 months of age the infant upper airway microbiota cluster into six compositionally distinct groups (MCS) typically dominated by *Moraxella, Streptococcus, Staphylococcus, Corynebacterium, Haemophilus*, or *Alloiococcus* [genus *Dolosigranulum* in some databases, (Bosch et al., 2016)]. As microbial complexity increases, microbiota colonization patterns arise and these have been shown to relate to the frequency of acute respiratory infection. More specifically, *Corynebacterium-, Staphylococcus-*, or *Alloiococcus-*dominated nasal microbiota were associated with reduced risk of respiratory illness, while *Streptococcus-, Moraxella-*, and *Haemophilus*-dominated microbiota associated with increased risk (Teo et al., 2015). Moreover, those children with early life colonization by *Streptococcus*-dominated microbiota were at increased risk of asthma development in childhood, indicating that early life microbial colonization of the airway mucosal surface is associated with long-term respiratory outcomes (Bosch et al., 2016). Environmental exposures such as antibiotic use, season, and presence of siblings were positively associated with *Haemophilus, Streptococcus*, and *Moraxella* MCS. Therefore, understanding the succession of microbial communities in the airways, both in childhood and as an adult, is paramount to our understanding of chronic airway disease.

Dysbiosis (altered) microbiota is a hallmark of CRS and other chronic respiratory diseases (Abreu et al., 2012; Biswas et al., 2015; Yu et al., 2015; Wagner Mackenzie et al., 2017). A recent meta-analysis of 11 studies from 170 CRS patients and 79 non-CRS controls demonstrated that, despite a large degree of interpersonal variation, CRS was associated with reduced bacterial diversity and fragmented ecological networks (Wagner Mackenzie et al., 2017). Another study of sinus microbiota in CRS patients demonstrated that at least four compositionally distinct sinus mucosal microbiota community states exist, each dominated by *Streptococcaceae, Pseudomonadaceae, Corynebacteriaceae*, or *Staphylococcaceae* and exhibiting a specific suite of co-associated lower abundance bacterial taxa (Cope et al., 2017). Of these four microbiota states, patients with *Corynebacteriaceae-*dominated communities exhibited significantly increased IL-5 gene expression and were at significantly higher risk for nasal polyps (Cope et al., 2017). This suggests that discrete host-microbiota interactions are associated with specific CRS phenotypes and opens the possibility of more targeted therapy e.g., anti-IL5 or specific probiotic taxa for subsets of CRS patients who exhibit *Corynebacterium*-dominated sinus mucosal microbiota and increased IL5.

## Interspecies microbe-microbe and microbe-host interactions

Emerging data in the field of microbiome research indicates that bacterial, fungal, and viral components of the airway microbiome do not exist in isolation but in complex communities that engage in microbe-microbe-host interactions. Microbes, including those that colonize mucosal surfaces of the human host, commonly reside in biofilms; complex surface-attached communities of mixed species microbes encased in a protective extracellular matrix (Lee and Yoon, 2017). This close contact facilitates microbe-microbe-host interactions including microbial metabolite cross-feeding (Ramsey et al., 2011) and quorum sensing; microbial derived inter-species signaling molecules (Waters and Bassler, 2005). Mixed fungal-bacterial biofilms have been comprehensively studied in the context of cystic fibrosis (Leclair and Hogan, 2010) but have also been observed in the sinuses of CRS patients (Healy et al., 2008; Foreman et al., 2009) as well as healthy individuals (Singh et al., 2015). However, little is known of the chemical lexicon that governs microbe-microbe-host interactions at the mucosal interface in humans *in situ*, through newer studies are addressing this new and exciting field.

Bacterial and fungal interspecies interactions play a critical role in shaping microbial community composition and behavior within a niche (Harriott and Noverr, 2009; Peters et al., 2012; Wolcott et al., 2013). In dental biofilms, microbes undergo spatio-temporal interactions in which an initial or pioneer bacterial species attaches to the tooth surface and alters ecosystem conditions to permit co-colonization by subsequent bacterial species (Colombo et al., 2013). In oral candidiasis, *Staphylococcus aureus* exhibits increased infectivity, biofilm development, and antibiotic resistance when detected in association with *Candida albicans* (Harriott and Noverr, 2009), indicating that co-associated microbes play an important role in modulating pathogenicity. In a murine model, data indicates that *S. aureus* selectively attaches to *C. albicans* hyphae, using this as a mechanism to penetrate oral tissue, enter the bloodstream and increase pathogenicity (Peters et al., 2012).

Interspecies interactions are also implicated in selection of community members within a niche. Nasopharyngeal microbiota undergoes succession with specific relatively simple pioneering microbiota typically dominated by *Staphylococcus* or *Corynebacterium* giving rise to communities of increasing complexity over the first year of life (Teo et al., 2015). These observations have important implications. First, they demonstrate that the composition of the microbiota in the upper airways influences the susceptibility to airway infection, supporting earlier murine studies by Abreu et al. (2012). Second, they implicate early life airway microbiota in the origins of chronic inflammatory airway disease. Hence an improved understanding of how specific upper airway microbiota, in particular those that protect against airway infection, develop is necessary. A recent study of the pediatric nasopharyngeal microbiota has begun to shed light on these processes. Using 16S rRNA gene sequencing of 200 pediatric nasal swabs paired with *in vitro* experimental methods, the authors demonstrated an inverse relationship between *Corynebacterium* and *Streptococcus* colonization. They also demonstrated direct antagonism between *Corynebacterium accolens* and *Streptococcus pneumoniae* mediated by *C. accolens* hydrolysis of human triacylglycerols to free fatty acids (FFAs). These FFAs inhibit *S. pneumoniae* growth and represent a molecular mechanism by which *Corynebacterium* may competitively exclude specific microbial species and presumably shape the nasopharyngeal microbiome (Bomar et al., 2016). Future studies examining how the key species in distinct CRS-associated mucosal microbiota cooperate to promote or prevent respiratory infection and chronic inflammatory disease development represents a ripe area for research, and one that will likely identify novel microbial-derived biologics for future clinical development.

### Role of quorum sensing systems in interspecies interactions: lessons from *ex vivo* and *in vitro* experiments

Microbial quorum sensing (QS) represents an important mode of microbial inter-species biochemical communication. QS molecules are small diffusible molecules produced by microorganisms (Rutherford and Bassler, 2012) that permit microbes to sense and respond to self or non-self microbial population densities. Once QS molecules reach a concentration threshold (quorum), they trigger specific gene expression profiles that alter the physiological state of the microbial cell in response to such signals. Several QS signaling molecules have been identified, including N-acyl-homoserine lactones (AHL) produced by gram-negative bacteria, oligopeptides produced by gram-positive bacteria, autoinducer-2 (AI-2) which may be produced by both Gram-positive and negative species, and farnesol, tyrosol, phenylethanol, and tryptophol produced by fungi (Fuqua and Greenberg, 2002; Juhas et al., 2005). Among the QS systems, AI-2 facilitates interspecies interactions and has been shown to induce polymicrobial biofilm formation by oral microbiota *Streptococcus oralis* and *Acinetobacter naeslundii* (Moons et al., 2009). QS systems not only sense population density but also regulate a variety of physiological traits, including spatial differentiation in biofilms (Lee et al., 2017), antibiotic resistance (Karatuna and Yagci, 2010), and expression of virulence factors. For example, a recently identified QS signal, diffusible signal factor (DSF), secreted by *Stenotrophomonas maltophilia* increased resistance to polymyxin and increased *P. aeruginosa* biofilm development *in vitro* (Moons et al., 2009; Tashiro et al., 2013). Microbial QS molecules also mediate host immune responses. *P. aeruginosa* C12 homoserine lactone (C12-HSL) rapidly induces apoptosis in airway epithelial cells (Schwarzer et al., 2012) representing a possible mechanism behind accumulation of apoptotic cells in the lungs of CF patients. In addition to C12-HSL, *Pseudomonas* quinolone signal (PQS) is a potent immune modulator; this QS molecule inhibits proliferation of *ex vivo* peripheral blood leukocytes and inhibits IL2 gene expression (Hooi et al., 2004). In CRS, C12-HSL and Gram-negative AHLs are potent agonists of bitter taste receptors (T2R38) present in the sinonasal epithelium activating NOS-dependent NO production (Cohen, 2017). Genetic polymorphisms T2R38 may underlie susceptibility to CRS or microbiome dysbiosis. These results suggest that QS molecules are important to microbial signaling but also at the host-microbe interface.

Perhaps the most comprehensively studied microbial interaction is between *C. albicans* and *P. aeruginosa* (Hogan and Kolter, 2002; Hogan et al., 2004; De Sordi and Mühlschlegel, 2009; Wongsuk et al., 2016), two microbes that frequently co-colonize in the CF lung. Normally, bacterial peptidoglycan-like molecules promote hyphae production by *C. albicans*, and a more pathogenic fungal phenotype. However, when *P. aeruginosa* selectively attaches to the hyphae of *C. albicans* and inhibits fungal growth by secreting several virulence factors such as pyocyanin, phospholipase C, and phenazine (Hogan et al., 2004; Tashiro et al., 2013). However, the interaction between *Pseudomonas* and *Candida* is not unidirectional; *C. albicans* can respond to a *P. aeruginosa* QS molecule (3-oxo-C12-HSL) and remain in yeast form to survive *P. aeruginosa* antagonism. Farnesol, a *C. albicans* QS molecule, represses PQS, which in turn regulates pyocyanin production, iron chelation, elastase production, and biofilm formation of *P. aeruginosa* (De Sordi and Mühlschlegel, 2009). The interactions between *C. albicans* and *S. aureus* are also of clinical interest; both species co-colonize various human mucosa including the sinonasal, lower respiratory, oral, vaginal, and urinary tract (Costa-Orlandi et al., 2017). Co-infection of murine oral mucosa with these two species is associated with enhanced virulence and resistance to host immune mechanisms, which may be mediated, at least in part by increased production of virulence factor and stress response proteins by these co-associated species (Costa-Orlandi et al., 2017).

### Other mechanisms of interspecies interactions

Inter-species metabolic cross-feeding also represents a means by which microbes cooperatively interact. For example, *Pseudomonas fluorescens* biofilm formation in milk results in its production of an extracellular matrix, which may be utilized by *Lactococcus lactis* to facilitate its biofilm formation. In return, *L. lactis* produces lactic acid that is serves as a nutritional substrate by *P. fluorescens* (Moons et al., 2009). In another example, an atypical *Escherichia coli* (ATEC) strain isolated from murine feces produces an excessive amount of catalase which catalyzes the decomposition of hydrogen peroxide to water and oxygen, a critical function that protects cells from oxidative damage by reactive oxygen species (ROS). Increased abundance of this ATEC strain in the GI tract is associated with increased colonization by ROS-sensitive bacteria such as *Vibrio cholera* (Yoon et al., 2016). Metabolic cross-feeding may be a mechanism for selection of co-colonizers in complex mucosal-associated microbial communities.

Co-colonizing viral and bacterial communities may influence disease pathogenesis through modulation of the host immune response or epithelial integrity. Infection with common respiratory viruses including influenza A virus, respiratory syncytial virus (RSV), adenovirus, and human rhinovirus can alter the physiology of airway epithelial cells and immune response of hosts and can lead to increased susceptibility to bacterial colonization (Ichinohe et al., 2011; Lynch, 2014). Viral particles may also bind to components of bacteria, such as lipopolysaccharide (LPS), prior to transmission to the human host. For example, poliovirus with the ability to bind LPS was more stable in the murine gut than a mutant virus with reduced LPS-binding (Robinson et al., 2014). In this case, presence of gram-negative bacteria in the GI tract supported poliovirus infectivity. Thus, manipulation of bacterial communities may represent a novel mode of anti-viral therapy. Supporting this, nasal administration of the bacteria *Lactobacillus rhamnosus* modulated levels of IFN-α, IFN-β, and IL-6, which was protective from subsequent RSV infection in a mouse model (Tomosada et al., 2013). Conversely pre-infection with RSV in the absence of specific protective microbiota can influence bacterial community composition and subsequent inflammatory response. A chinchilla model of otitis media demonstrated that animals infected with RSV had reduced β-defensin-1 gene expression, an antimicrobial peptide (AMP). In this model, co-infection of nontypeable *Haemophilus influenza* (NTHi) and RSV results significantly higher load of NTHi from nasopharyngeal fluid when compared to the mono-species infection (McGillivary et al., 2009). Thus, disruption of a single AMP by viral infection led to increased susceptibility to colonization by an airway pathobiont. These studies provide evidence that the viral-bacterial interaction through immunomodulaton of the host are important to pathogenesis or development of airway diseases (Lynch, 2014; Lynch et al., 2017) and suggest that healthy sinonasal microbiota may protect from viral infection.

## Challenges and future directions in our understanding of mucosal microbial communities

**Figure 1:**
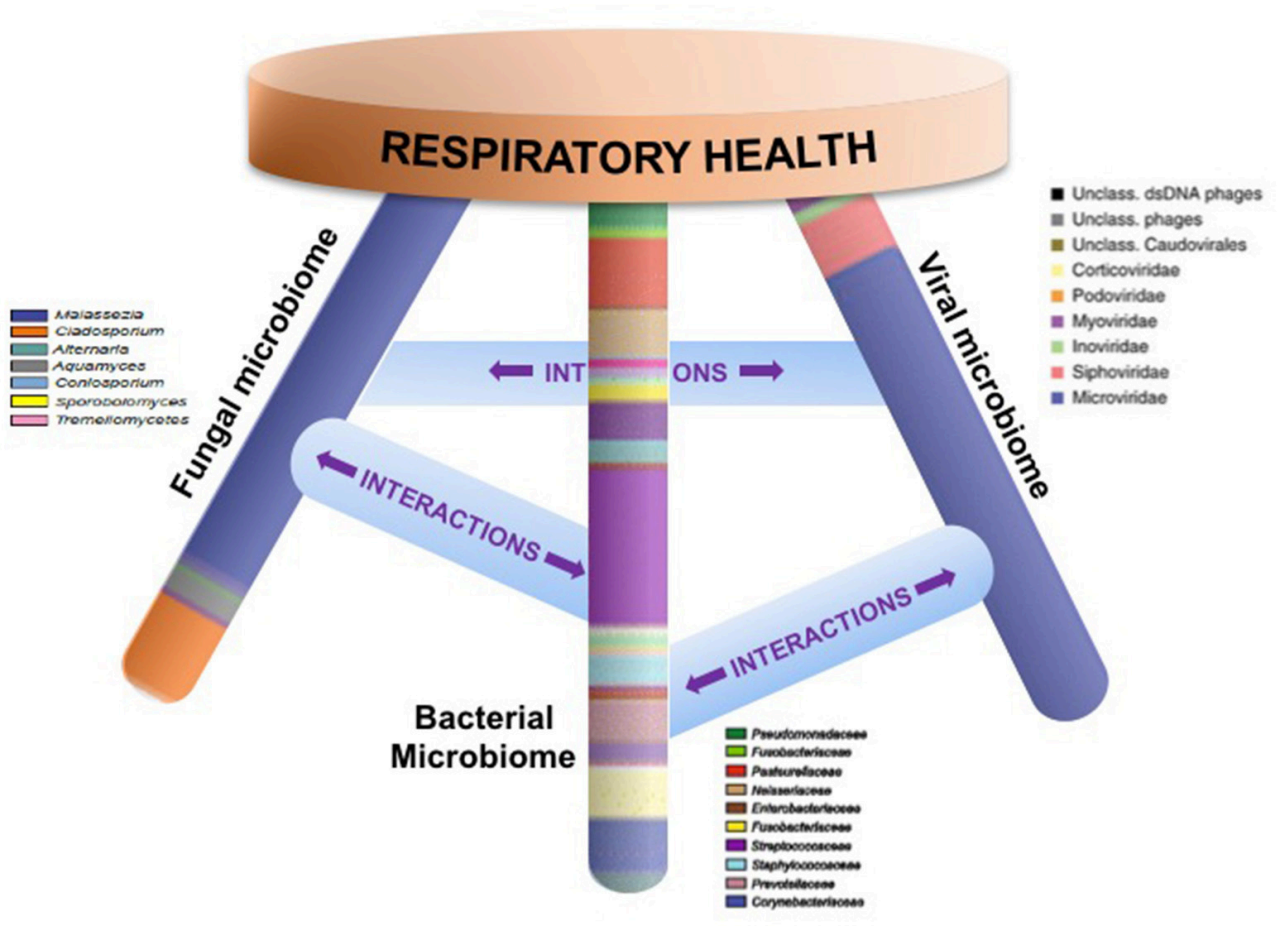
Respiratory health is likely affected by homeostasis of bacterial, fungal, and viral communities and their specific interactions. Studies that elucidate these complex interactions are necessary in order to design specific therapeutics that target microbial community dysbiosis in the airways.

An emerging body of work thus indicates that local mucosal microbiota composition is related to host immune response (Sommer and Backhed, 2013; Jain et al., 2016). These immunomodulatory effects may be due to complex microbial interactions within mixed-species microbial communities or molecular cross-talk and inter-species signaling between microbial communities and the host (Figure 1). Mechanistic evidence from studies of the oral or gut microbiome can form a framework for similar studies of the sinonasal microbiome-host interface. Layering multiple ‘omic assays may guide more specific hypotheses that can be experimentally validated *in vitro* and in animal or *ex vivo* models to understand how complex interactions drive inflammation. These studies will present challenges, but improvements in culturing techniques and advances in non-animal human cell culture or biomimetic devices that replicate mechanistic and functional aspects of organs will likely drive studies of the host-microbiome interface forward to new and exciting directions. Ultimately, understanding the succession of the microbiota in the airways and the immunological consequences of the observed heterogeneity in CRS-associated microbiota and immune response will lead to the development of strategies to rationally manipulate these communities toward a healthy state.

## Author contributions

KL drafted and revised the manuscript. EC, SL, and SP conceived, wrote, and revised the manuscript. AG conceived and revised the manuscript.

### Conflict of interest statement

The authors declare that the research was conducted in the absence of any commercial or financial relationships that could be construed as a potential conflict of interest. The reviewer AW and handling Editor declared their shared affiliation.
